# Arachnoid Cyst and Psychosis

**DOI:** 10.7759/cureus.1707

**Published:** 2017-09-21

**Authors:** Afaque H Khan, Syed E Ahmed

**Affiliations:** 1 Psychiatry, Heartland Behavioral Healthcare, Department of Mental Health and Addiction, State of Ohio, Northeast Ohio Medical University; 2 Heartland Behavioral Healthcare, Department of Mental Health and Addiction, State of Ohio, Northeast Ohio Medical University

**Keywords:** arachnoid cyst, psychosis, schizoaffective disorder bipolar type

## Abstract

Few case reports discuss suspected psychiatric disturbances secondary to arachnoid cysts. To the best of our knowledge, psychosis from arachnoid cysts has not been reported in the recent literature. This case report discusses our experience with a 55-year-old male patient who manifested multiple psychotic episodes suspected in association with an arachnoid cyst. During the initial examinations, a computed tomography (CT) scan of his head revealed a relatively large left anterior superior arachnoid cyst with a mass effect on the frontal lobe. Despite multiple trials of combinations of mood stabilizers and psychotropic medications, the patient continued to deteriorate. We ordered a repeat CT of his head along with a magnetic resonance image and consulted a neurosurgeon for a possible surgical intervention. The neurosurgeon concluded that the patient’s psychiatric symptoms were unrelated to the arachnoid cyst. While we were unable to determine the etiology of our patient’s psychosis, we believe that the arachnoid cyst played at least some part, as evidenced by reports in the literature. Future advancements in radiological technology may allow for a better exploration of the causal relationship between psychiatric illnesses and brain lesions.

## Introduction

 Arachnoid cysts are rare benign neurological tumors, and few exhibit any symptoms. Their known incidence accounts for only 1% of all intracranial space-occupying lesions, and detection is usually incidental. Case reports regarding the coexistence of arachnoid cysts with psychiatric disorders are sparse [1]. Arachnoid cysts are formed when the arachnoid membrane contains cerebrospinal fluid and are frequently located in the Sylvian fissure [2], middle cranial fossa, suprasellar area, or posterior fossa. Clinical manifestations are unpredictable, and vary from quiescence to active symptoms and spontaneous disappearance [3]. A recent surge of interest in arachnoid cysts and possible associations with psychosis has raised questions about etiology and adequate therapeutic approaches.

## Case presentation

**Figure 1:**
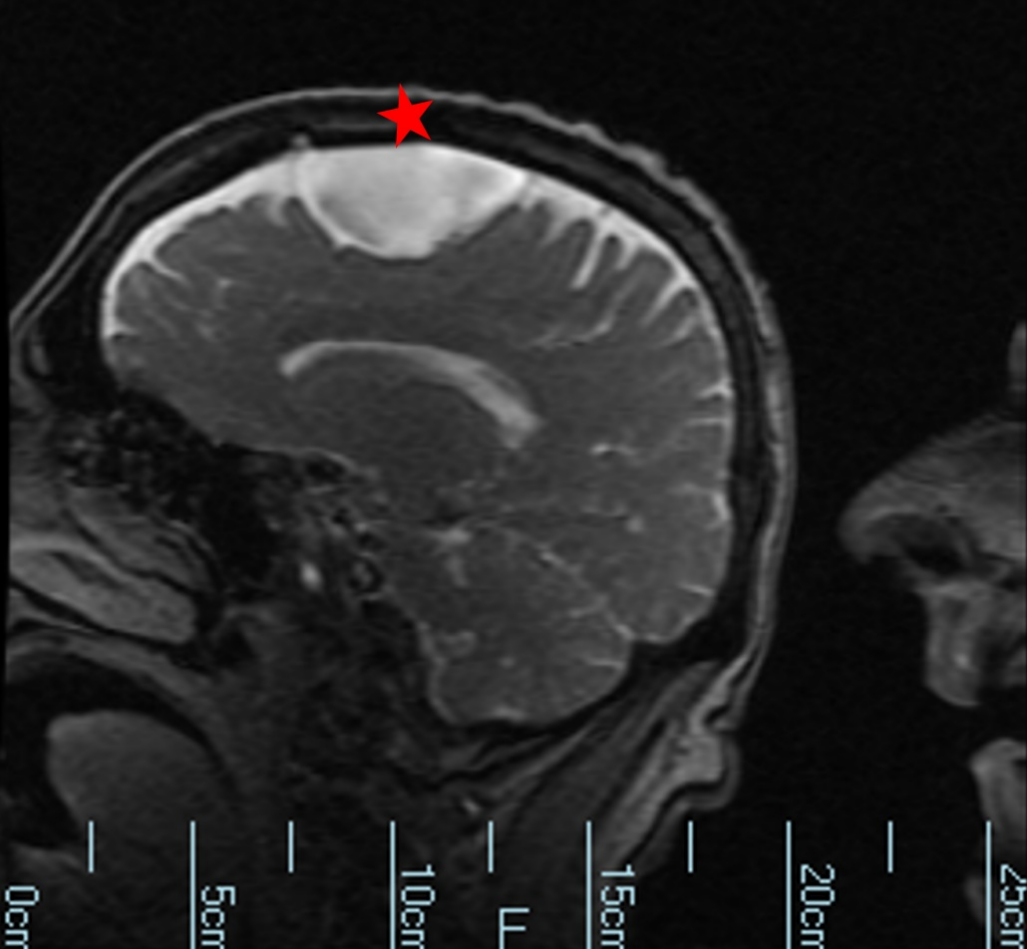
Computed tomography showing a 4.3 x 5.6 x 3.1 cm cerebrospinal fluid density structure over the left frontal convexity with mass effect on the adjacent frontal lobe and slight scalloping of the overlying calvarium. This area suppresses completely on fluid-attenuated inversion recovery imaging (FLAIR) and demonstrates no enhancement on post-contrast imaging. There is no abnormal signal intensity in the adjacent brain parenchyma. Cyst is denoted by a red star.

**Figure 2:**
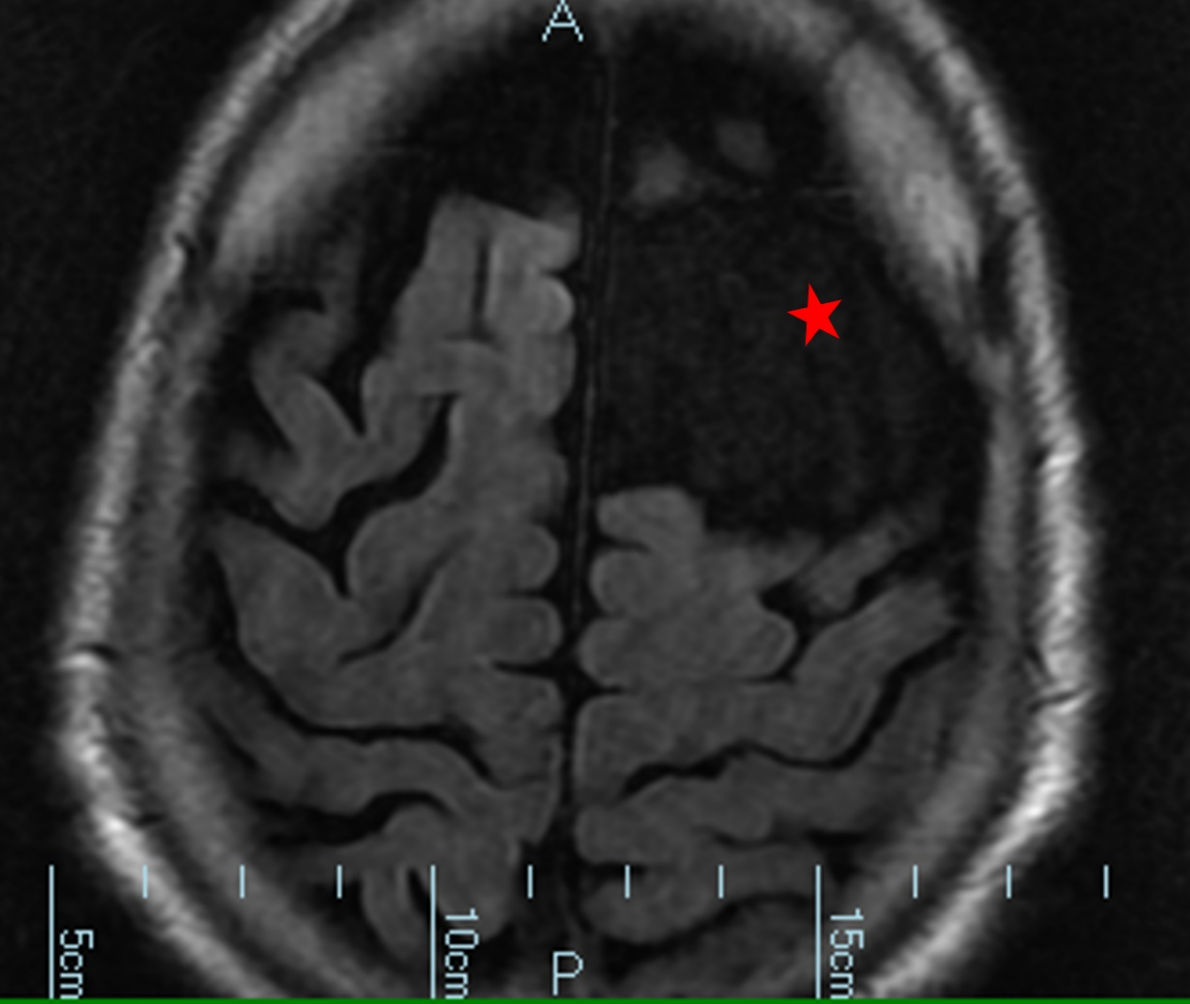
Computed tomography showing arachnoid cyst of the left frontal lobe. Cyst is denoted by a red star.

A 55-year-old Caucasian man was admitted to the psychiatric state hospital from the emergency department with sudden onset of agitation, aggression, insomnia, hypersexuality, auditory, visual hallucinations, and persecutory delusions for the past four days. He had a long history of Schizoaffective Disorder Bipolar type 1 according to the Diagnostic and Statistical Manual of Mental Disorders (DSM-V) criteria. The patient had been stable for the past 17 years with the help of mood stabilizers and psychotropic medications, but six months prior to admission, he experienced frequent relapses despite good compliance and treatment adherence. On admission, the patient exhibited symptoms of racing thoughts, flight of ideas, insomnia for the past four days, as well as visual and auditory hallucinations. Additionally, he displayed extreme sexual preoccupation, paranoid delusions, and pressured speech. While in the emergency department, a computed tomography (CT) scan of his head revealed a relatively large left anterior superior arachnoid cyst with a mass effect on the frontal lobe (Figure 1, Figure 2).

This was the most recent of multiple emergency department admissions where a brief period of stabilization was achieved with antipsychotic medications and mood stabilizers. He had oscillated between local hospital emergency departments and the crisis stabilization unit due to repeated decompensation. The presence of acute, intense, and erratic symptoms led to his admission to the state psychiatric facility.

A physical examination on admission to the psychiatric hospital showed no neurological deficit, and the patient’s attention, concentration, and memory were unimpaired. The mental status evaluation score was 27/30. Routine laboratory test results including complete blood count, comprehensive metabolic panel, and thyroid panel were all unremarkable. Also, a nonreactive rapid plasma reagin test for syphilis and non-detectable HIV antibodies ruled out an infectious etiology. 

For control of his psychotic symptoms, the patient was given trials of multiple psychotropics, including Ziprasidone, Paliperidone, Quetiapine, and Aripiprazole along with Clonazepam for agitation. Valproic acid and Oxcarbazepine were added to ameliorate his labile mood. We noted a mild improvement in his mood and agitation, but his clinical condition remained largely unchanged.

**Figure 3:**
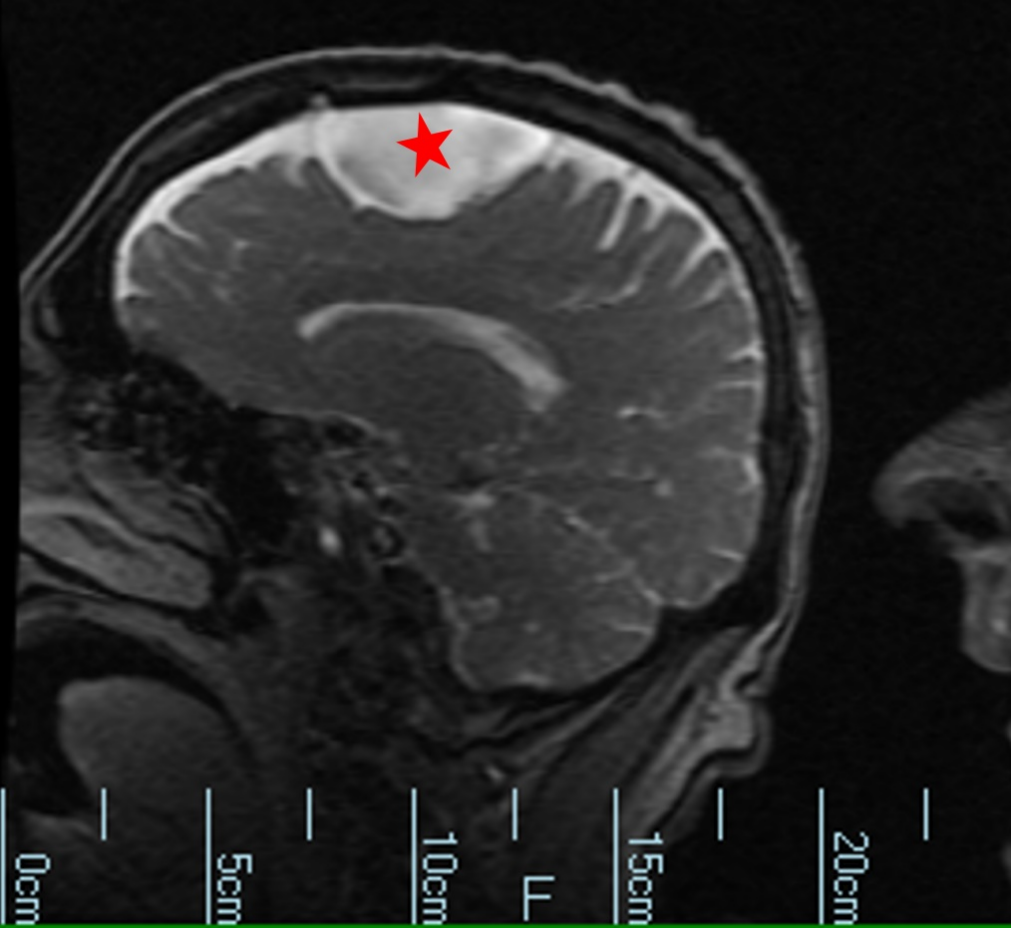
Computed tomography showing arachnoid cyst on left frontal convexity with mass effect on frontal lobe. Cyst is denoted by a red star.

**Figure 4:**
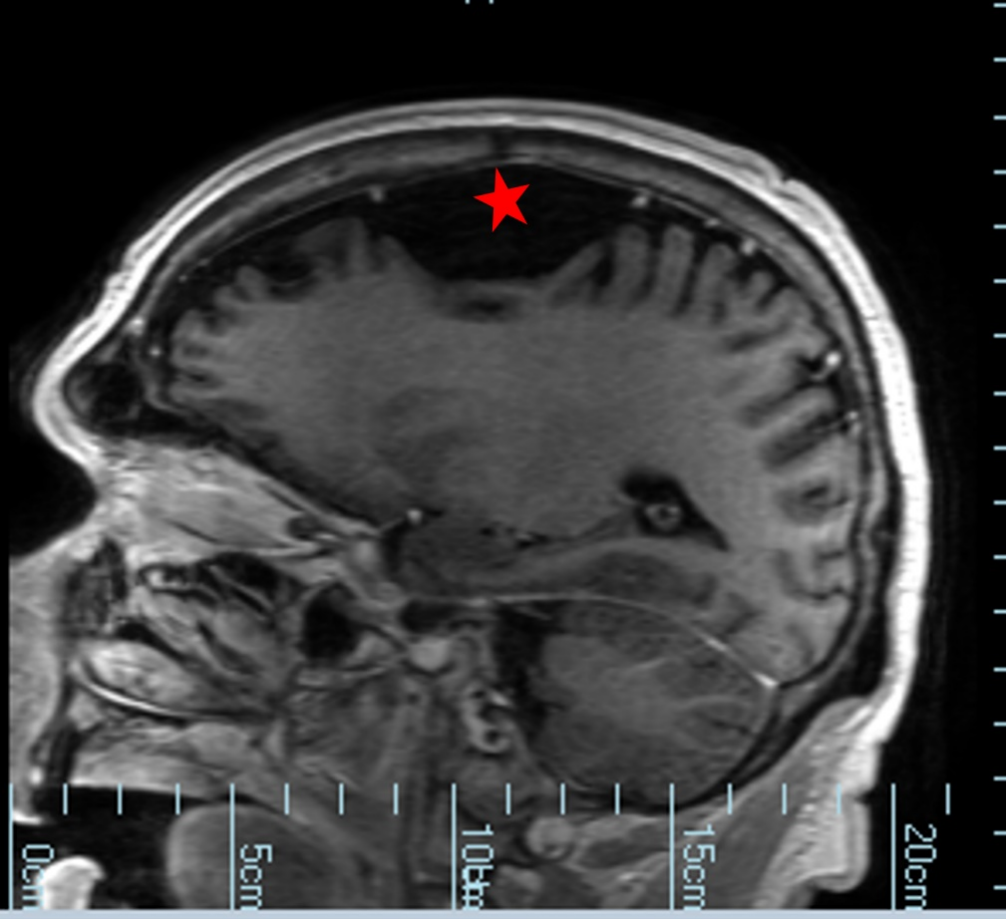
Magnetic resonance imaging without contrast. Cyst is denoted by a red star.

**Figure 5:**
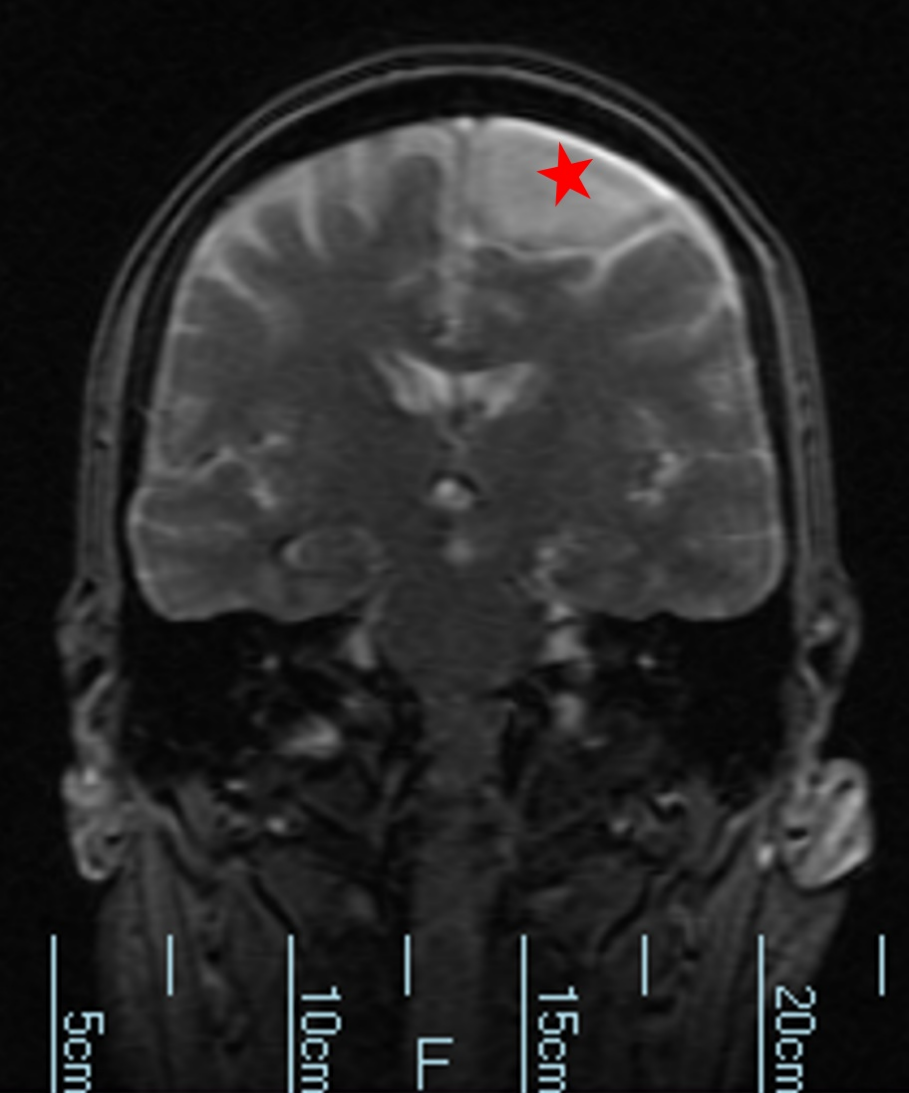
Magnetic resonance imaging without contrast and diffusion imaging shows no hyperacute, acute or early subacute infarction. No abnormal parenchymal or leptomeningeal enhancement. Cyst is denoted by a red star.

Given the atypical course of the illness, we sought a neurosurgery consultation and obtained a second CT scan of the head and a magnetic resonance image (MRI) of the brain. The CT scan revealed an arachnoid cyst above the left frontal lobe measuring 5.8 cm x 4.7 cm x 2.6 cm with diffuse age-related mild cerebral atrophy (Figure 3). These findings were unchanged from previous reports. Similar findings were also noted on the MRI, which revealed a 5.6 cm arachnoid cyst on the left frontal convexity with a mass effect on the frontal lobe, without parenchymal hemorrhage (Figure 4, Figure 5). On neurological exam, we found no signs of intracranial pressure such as papilledema or focal neurological deficits. The neurosurgical consultant concluded the radiologic finding was likely an incidental congenital finding, and that the patient’s psychiatric symptoms were unrelated to the presence of the arachnoid cyst. Therefore, conservative management was proposed.

## Discussion

This case report explores the possible causal relationship between an arachnoid cyst and psychotic symptoms [2]. Is it possible our patient’s brain lesion precipitated psychiatric symptoms or was the lesion an incidental finding, benign and unrelated to his psychiatric presentation? Despite lacking the development history of his cystic lesion, we can say the patient's abnormal behavior was likely secondary to the arachnoid cyst based on the following reasons: (i) the patient had been stable for 17 years prior to the radiological discovery of the cyst; (ii) he experienced multiple admissions and relapses despite good compliance with higher doses of psychotropic and mood stabilizing medications in the inpatient and outpatient settings; (iii) laboratory investigations yielded no relevant clinical findings; (iv) the mass effect of an arachnoid cyst on the frontal lobe and surrounding structures may cause psychotic symptoms [2-3]. Baquero, et al. underscored the importance of considering an arachnoid cyst as the inciting cause of psychopathological symptoms, even psychosis, and such symptoms can be the only clinical manifestation of the cyst [4]. Their report describes the termination of symptoms by neurosurgical removal of the large arachnoid cyst.

Our patient only achieved mild and transient stability with trials of multiple antipsychotic and mood stabilizers. This raises increased suspicions of an organic etiology of his disease course. The counterargument to this consideration is the patient could be following the natural longitudinal course leading to rapid cycling bipolar disorder. In the DSM-V, rapid cycling bipolar disorder is characterized by at least four mood episodes during a 12-month period [5]. Rapid cycling bipolar patients are unresponsive to all medications useful to non-rapid cycling patients [6]. Our patient’s presentation of symptoms and disease course was contrary to that expected for rapid cycling bipolar disorder. During a period of two years, he had over 12 admissions to the emergency department and crisis stabilization unit, and three hospitalizations to the state psychiatric hospital. Maximal doses of antipsychotic and various mood stabilizers yielded an ineffective response. This further supports our argument that the cause of psychosis may be an organic etiology.

## Conclusions

It is difficult to conclude whether the arachnoid cyst was asymptomatic or responsible for the patient’s overall psychiatric symptoms due to the limited history of the development of the cystic lesion. However, the serious changes to the left frontal lobe and the presence of neuropsychological symptoms suggest the reasonable conclusion that the lesion plays some part in the etiology. The typical symptoms, paranoid delusions, extreme sexual preoccupation, insomnia, and violent behavior may have an organic cause if the patient shows poor response to treatment. While the neurosurgical consultation concluded that our patient’s psychiatric symptoms were unrelated to the arachnoid cyst, the literature presents evidence of improvement and resolution of psychosis after neurosurgical removal of an arachnoid cyst. We are optimistic that further advancements in radiological technology will enable a better exploration of the potential causal relationship between psychiatric illnesses and organic brain lesions. Such advances would enhance the reliability and precision of both diagnosis and treatment. More research is required to optimize the therapeutic approach for patients with arachnoid cysts and concurrent psychosis.
